# Supplementary material for: The Systems Biology Research Tool: evolvable open-source software
Source: BMC Syst Biol. 2008 Jun 29;2:55. doi: 10.1186/1752-0509-2-55 (PMC2446383; doi:10.1186/1752-0509-2-55)
Supplement: Additional file 1 — SBRT Archive. An archive of the current version of the Systems Biology Research Tool. [file 1752-0509-2-55-S1.zip › sbrt-1.4.0/doc/developers_guide/api/sbrt/shell/text/AbstractMapEntryFormat.html]

AbstractMapEntryFormat


|  |  |  |
| --- | --- | --- |
| **Overview** \| **Package** \| **Class** \| **Use** \| **Tree** \| **Deprecated** \| **Index** \| **Help** | |  |
| **PREV CLASS**   **NEXT CLASS** | **FRAMES**    **NO FRAMES**     **All Classes** |  |
| SUMMARY: NESTED \| FIELD \| CONSTR \| METHOD | DETAIL: FIELD \| CONSTR \| METHOD |  |


---


## sbrt.shell.text Class AbstractMapEntryFormat<K,V>

```
java.lang.Object
  sbrt.shell.text.AbstractMapEntryFormat<K,V>
```

**Type Parameters:**: `K` - the key type.: `V` - the value type.

**All Implemented Interfaces:**: Format, MapEntryFormat<K,V>

**Direct Known Subclasses:**: ColonMapEntryFormatV1, EqualsMapEntryFormatV1

---

``` public abstract class AbstractMapEntryFormat<K,V> extends java.lang.Object implements MapEntryFormat<K,V> ```

This abstract class is a skeleton implementation of
`MapEntryFormat`. A concrete class with a definite
format can be created by simply overriding the
constructor of this class.

**Author:**
:   This class was written and documented by
    Jeremiah Wright while in the Wagner lab.

---

**Constructor Summary**

`AbstractMapEntryFormat(java.lang.String delimiter)`
  
          Constructs a new map entry format using the provided delimiter
and zero-length strings as left and right paddings.

`AbstractMapEntryFormat(java.lang.String leftPadding,
java.lang.String delimiter,
java.lang.String rightPadding)`
  
          Constructs a new map entry format from the provided arguments.


| **Method Summary** | |
| --- | --- |
| `java.lang.String` | `formatPair(java.util.Map.Entry<? extends K,? extends V> entry)`             Returns a formatted string representation of the provided map entry. |
| `<S extends K,T extends V> java.lang.String` | `formatPair(S key, T value)`             Returns a string representation of the provided key-value pair. |
| `java.lang.String` | `getDelimiter()`             Returns the delimiter used to separate the key from the value in a key-value pair. |
| `java.lang.String` | `getLeftPadding()`             Returns the left padding used to create formatted string representations of key-value pairs. |
| `java.lang.String` | `getRightPadding()`             Returns the right padding used to create formatted string representations of key-value pairs. |
| `java.util.Map.Entry<K,V>` | `parsePair(java.lang.String mapEntry)`             Parses the provided map entry string, and returns its corresponding map entry. |

**Methods inherited from class java.lang.Object**

`clone, equals, finalize, getClass, hashCode, notify, notifyAll, toString, wait, wait, wait`

**Methods inherited from interface sbrt.shell.text.MapEntryFormat**

`formatKey, formatValue, parseKey, parseValue`

**Constructor Detail**

### AbstractMapEntryFormat

```
public AbstractMapEntryFormat(java.lang.String leftPadding,
                              java.lang.String delimiter,
                              java.lang.String rightPadding)
```

:   Constructs a new map entry format from the provided arguments.

    **Parameters:**: `leftPadding` - a string of whitespace characters placed between the key and delimiter when producing a formatted string representation of a map entry.: `delimiter` - the delimiter used to separate key-value pairs when producing and parsing a formatted string representation of a map entry.: `rightPadding` - a string of whitespace characters placed between the delimiter and value when producing a formatted string representation of a map entry. **Throws:**: `java.lang.IllegalArgumentException` - if the left or right paddings contain non-whitespace characters.: `java.lang.NullPointerException` - if any provided argument is `null`.: `java.util.regex.PatternSyntaxException` - if the provided delimiter cannot be compiled into a regular expression.

---


### AbstractMapEntryFormat

```
public AbstractMapEntryFormat(java.lang.String delimiter)
```

:   Constructs a new map entry format using the provided delimiter
    and zero-length strings as left and right paddings.

    **Parameters:**: `delimiter` - the delimiter used to separate key-value pairs when producing and parsing a formatted string representation of a map entry. **Throws:**: `java.lang.NullPointerException` - if the provided argument is `null`.: `java.util.regex.PatternSyntaxException` - if the provided delimiter cannot be compiled into a regular expression. **See Also:**: `AbstractMapEntryFormat(String, String, String)`


**Method Detail**

### getDelimiter

```
public final java.lang.String getDelimiter()
```

:   Returns the delimiter used to separate the key from the
    value in a key-value pair.

    :   **Returns:**: the delimiter used to separate the key from the value in a key-value pair.

---


### getLeftPadding

```
public final java.lang.String getLeftPadding()
```

:   Returns the left padding used to create formatted
    string representations of key-value pairs. The left
    padding is inserted between the key and delimiter.
    In other words, it appears to the left of the delimiter
    in key-value strings.

    :   **Returns:**: the left padding used to create formatted string representations of key-value pairs.

---


### getRightPadding

```
public final java.lang.String getRightPadding()
```

:   Returns the right padding used to create formatted
    string representations of key-value pairs. The right
    padding is inserted between the delimiter and value.
    In other words, it appears to the right of the delimiter
    in key-value strings.

    :   **Returns:**: the right padding used to create formatted string representations of key-value pairs.

---


### formatPair

```
public <S extends K,T extends V> java.lang.String formatPair(S key,
                                                             T value)
```

:   Returns a string representation of the provided
    key-value pair.

    :   **Specified by:**: `formatPair` in interface `MapEntryFormat<K,V>`
    :   **Type Parameters:**: `S` - a type extending the primary key type.: `T` - a type extending the primary value type. **Parameters:**: `key` - the key.: `value` - the value. **Returns:**: a string representation of the provided key-value pair.

---


### formatPair

```
public java.lang.String formatPair(java.util.Map.Entry<? extends K,? extends V> entry)
```

:   Returns a formatted string representation of the
    provided map entry.

    :   **Specified by:**: `formatPair` in interface `MapEntryFormat<K,V>`
    :   **Parameters:**: `entry` - the map entry. **Returns:**: a formatted string representation of the provided map entry.

---


### parsePair

```
public java.util.Map.Entry<K,V> parsePair(java.lang.String mapEntry)
```

:   Parses the provided map entry string, and
    returns its corresponding map entry. The string is split on the
    *first* occurrence of the delimiter.

    :   **Specified by:**: `parsePair` in interface `MapEntryFormat<K,V>`
    :   **Parameters:**: `mapEntry` - the string representation of a single map entry. **Returns:**: a singleton map corresponding to the provided string.


---


|  |  |  |
| --- | --- | --- |
| **Overview** \| **Package** \| **Class** \| **Use** \| **Tree** \| **Deprecated** \| **Index** \| **Help** | |  |
| **PREV CLASS**   **NEXT CLASS** | **FRAMES**    **NO FRAMES**     **All Classes** |  |
| SUMMARY: NESTED \| FIELD \| CONSTR \| METHOD | DETAIL: FIELD \| CONSTR \| METHOD |  |


---
